# Supplementary figures and images for: Modeling Climate Change Effects on Genetic Diversity of an Endangered Horse Breed Using Canonical Correlations
Source: Animals (Basel). 2024 Feb 20;14(5):659. doi: 10.3390/ani14050659 (PMC10931216; doi:10.3390/ani14050659)

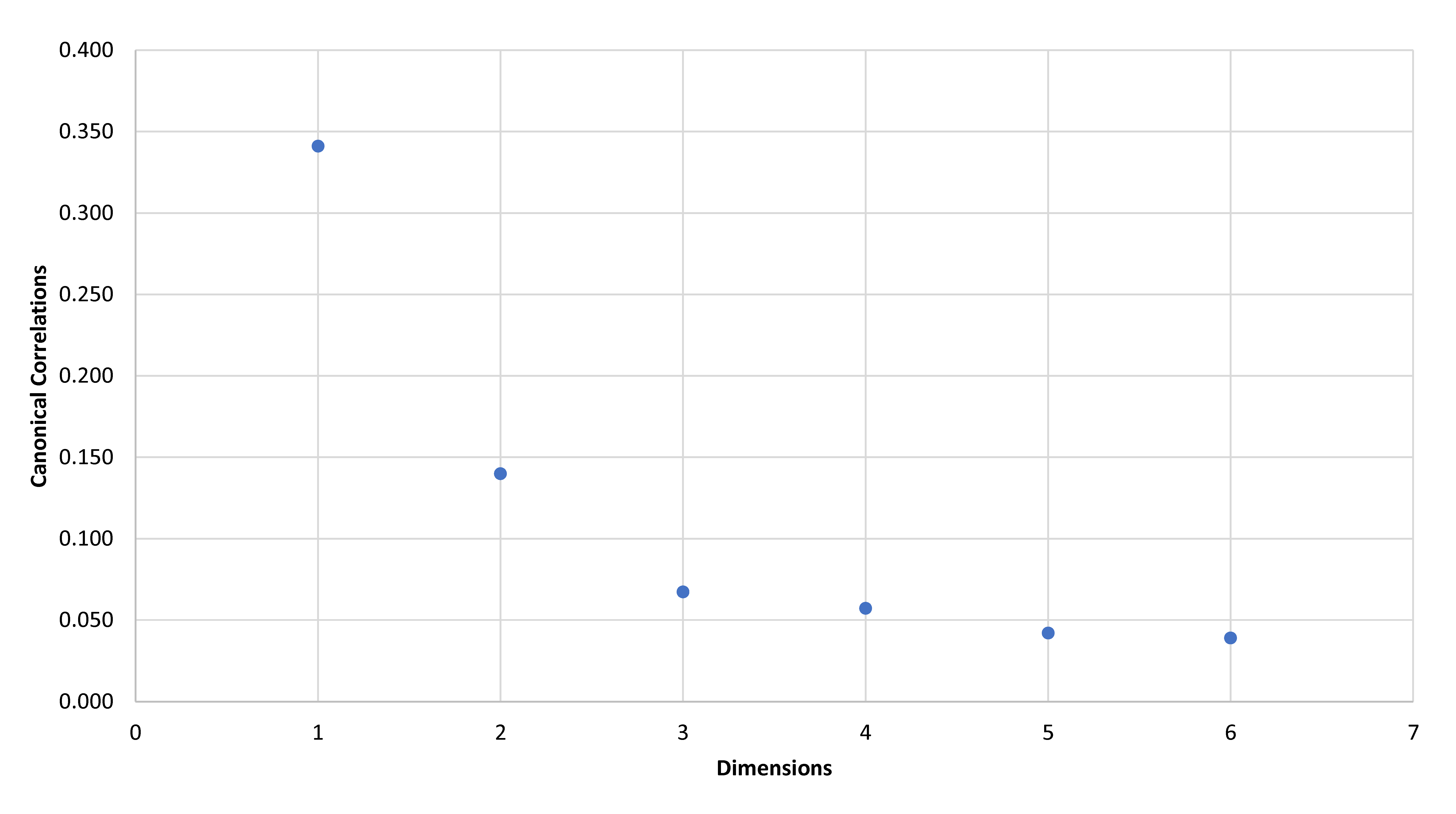

Supplement: Supplementary file 1 [file animals-14-00659-s001.zip › Supplementary Figure 1.tiff]
